# The complete chloroplast genome sequence of * Morus cathayana* and *Morus multicaulis*, and comparative analysis within genus *Morus* L

**DOI:** 10.7717/peerj.3037

**Published:** 2017-03-08

**Authors:** Wei Qing Kong, Jin Hong Yang

**Affiliations:** Shaanxi Key Laboratory of Sericulture, Ankang University, Ankang, Shaanxi, China

**Keywords:** *Morus cathayana*, *Morus multicaulis*, Mutation, Chloroplast genome, Codon usage

## Abstract

Trees in the Morus genera belong to the Moraceae family. To better understand the species status of genus *Morus* and to provide information for studies on evolutionary biology within the genus, the complete chloroplast (cp) genomes of *M. cathayana* and *M. multicaulis* were sequenced. The plastomes of the two species are 159,265 bp and 159,103 bp, respectively, with corresponding 83 and 82 simple sequence repeats (SSRs). Similar to the SSRs of *M. mongolica* and *M. indica* cp genomes, more than 70% are mononucleotides, ten are in coding regions, and one exhibits nucleotide content polymorphism. Results for codon usage and relative synonymous codon usage show a strong bias towards NNA and NNT codons in the two cp genomes. Analysis of a plot of the effective number of codons (ENc) for five *Morus* spp. cp genomes showed that most genes follow the standard curve, but several genes have ENc values below the expected curve. The results indicate that both natural selection and mutational bias have contributed to the codon bias. Ten highly variable regions were identified among the five *Morus* spp. cp genomes, and 154 single-nucleotide polymorphism mutation events were accurately located in the gene coding region.

## Introduction

Mulberry (genus *Morus*, family Moraceae) is widely distributed in Asia, Europe, North and South America, and Africa. The trees are cultivated in East, Central, and South Asia for domesticated silkworm and silk production, which is of economic importance. Some mulberry species are also valued for their hard wood, delicious fruit, bark for paper production, and multiple uses in traditional oriental medicine (Asano et al., 1994; Kim et al., 1999). Linnaeus classified the Moraceae family into order Urticales in 1753 using morphological characteristics. However, the Moraceae family has been repositioned into order Rosales on the basis of the phylogenetic relationships among some nuclear genes and chloroplast (cp) loci or genomes (Kong & Yang, 2016; Su et al., 2014; Zhang et al., 2011). *Morus* is a genus of trees in family Moraceae, and species classification within *Morus* is the subject of ongoing controversy. In the Annals of Mulberry Varieties in China (The Sericultural Research Institute Chinese Academy of Agricultural Sciences, 1993), more than 3,000 mulberry germplasm resources were classified into 15 species and four varieties. In the *Flora of China* (Zhou & Gilbert Michael, 2003), *Morus* is classified into two groups and 11 species.

With the development of sequencing technology in recent years, in addition to nuclear genome sequences, cp genes (*matK* and *rbcl*), gene spacer regions (*trnH*–*psbA*, *atpB*–*rbcL*, *trnC*–*ycf6*, and *trnL*–F), and cp genome information have been used to study plant molecular systematics (CBOL Plant Working Group, 2009; China Plant BOL Group et al., 2011; Kress & Erickson, 2007; Roy et al., 2010). A nuclear gene internal transcribed spacer (ITS) and three cp DNA fragments in a total of four sequences are generally recommended for investigation of near-edge species. In *Morus*, Zhao et al. (2005) used the ITS and *trnL*-F sequence to cluster 13 mulberry genotypes, representing nine species and three varieties, into five branches. There are no other reports of molecular systematics applicable to the differentiation and classification of *Morus* species.

Chloroplasts are photosynthetic organelles that occur widely in algae and plants. Although the cp genome is more conserved than the nuclear genome, many mutation events such as indels, substitutions, and inversions have been identified in cp DNA sequences (Ingvarsson, Ribstein & Taylor, 2003). As a DNA polymorphism index, indels and single-nucleotide polymorphisms (SNPs) can be used at a low taxonomic level. The DNA polymorphism rate between cp genomes was 0.15% for *M. yunnanensis* and *M. balansae*, 0.3% for *M. indica* and *M. mongolica* (Kong & Yang, 2016; Song et al., 2015), and 0.2% among four different Chinese ginseng strains (Zhao et al., 2015). These results indicate the presence of variable characteristics among the cp genomes of different species.

Complete cp sequences can provide insight into evolution and natural selection, and have been used in significant contributions concerning evolutionary mechanisms for species and chloroplasts (Asheesh & Vinay, 2012). To date, cp genomes have been reported for three *Morus* species: *M. mongolica*, *M. indica*, and *M. notabilis* (Chen et al., 2016; Kong & Yang, 2016; Ravi et al., 2006). However, there has been no research on codon biology or genome evolution. Here, we report the cp genomes for another two *Morus* species (*M. cathayana* and *M. multicaulis*) and compare the codon usage bias (CUB), sequence divergence, and mutation events among the five *Morus* spp. cp genomes (Table 1).

## Materials & Methods

### Sample collection, DNA extraction, and sequencing

Samples of *M. cathayana* were collected from the Qinba Mountain Area and *M. multicaulis* was acquired from Shandong Sericultural Research Institute. Both plants used in the study are now maintained in the mulberry field of Shaanxi Key Laboratory of Sericulture, Ankang University, China. The cp DNA was extracted from 10 g of fresh leaves using a modified high-salt method (Shi et al., 2012) and treated according to a standard procedure (Bartram et al., 2011) for sequencing on an Illumina Hiseq 2000 platform (2 * 125 bp).

**Table 1:** Summary of the features of *Morus* chloroplast genomes.

Species	GenBank No.	Genome size/GC content	LSC size/GC content	SSC size /GC content	IR size/GC content
*M. cathayana*	KU981118	159,265/36.16	88,143/33.77	19,844/29.20	25,639/42.95
*M. multicaulis*	KU981119	159,103/36.19	87,940/33.82	19,809/29.26	25,677/42.91
*M. indica*	NC_008359	158,484/36.37	87,386/34.12	19,742/29.35	25,678/42.92
*M. mongolica*	KM491711	158,459/36.29	87,367/33.97	19,736/29.33	25,678/42.92
*M. notabilis*	NC_027110	158,680/36.36	87,470/34.11	19,776/29.34	25,717/42.89

### Chloroplast genome assembly and annotation

Both reads were assembled using SOAP *de novo* software (Luo et al., 2012) with the Kmer and maximal read length set to 60 and 50, respectively. The resulted contigs were aligned to the *M. mongolica* cp genome as the reference genome. Ambiguous sequences were manually trimmed. The mean fold coverage of the final assembled *M. cathayana* and *M. multicaulis* cpDNA reached approximately 690- and 770-fold, respectively. Transfer RNAs (tRNAs), ribosomal RNAs (rRNAs), and protein-coding genes (PCGs) in the two cp genomes were annotated using Dual Organellar GenoMe Annotator (DOGMA) software (Wyman, Jansen & Boore, 2004). The tRNAs and their corresponding structures were further verified and predicted using the tRNAscan-SE 1.21 program (Schattner, Brooks & Lowe, 2005). The physical maps were drawn using the web tool Organellar Genome DRAW (OGDraw) v1.2 (Lohse, Drechsel & Bock, 2007).

### Analysis of simple sequence repeats (SSRs) and long repeat sequences

SSRs in *M. cathayana* and *M. multicaulis* cp genomes were detected using MISA (Thiel et al., 2003) with the minimal repeat number set to 10, 5, 4, 3, 3, and 3 for mono-, di-, tri-, tetra-, penta-, and hexa-nucleotides, respectively. The distribution, nucleotide composition, and polymorphism among SSRs were investigated. For long repeat sequences in five *Morus* species, the program REPuter was used to assess the number and location of all four type of forward match (F), reverse match (R), complement match (C) and palindromic match (P) (Kurtz et al., 2001). The identity of the repeat was limited to greater than 90% and the size was no less than 25 bp, respectively.

### Indices of codon usage

The amino acid composition and relative synonymous codon usage (RSCU) values for *M. cathayana* and *M. multicaulis* cp genomes were calculated using Mega 5.05 (Tamura et al., 2011). Then, the GC content of the first, second, and third codon positions (GC1, GC2, and GC3, respectively) and the overall GC content of the coding regions (GCc) were calculated manually. GC3S is the GC content at the third synonymously variable coding position excluding Met, Trp, and three stop codons. The CodonW program on the Mobyle server (http://www.mobyle.fr) was used to calculate the GC3s and the effective number of codons (ENc). An ENc plot (ENc vs GC3s) was also analyzed.

### Sequence divergence and mutation events analysis

A sliding window analysis was conducted using DnaSP 5.0 software (Librado & Rozas, 2009) for comparative analysis of the sequence divergence (Pi) among the cp genomes of *M. cathayana*, *M. multicaulis*, *M. mongolica*, *M. indica*, and *M. notabilis*. The window length was 600 bp and the step size was 200 bp.

To identify differences in coding sequences and corresponding amino acids among the five *Morus* cp genomes, pairwise alignment of the nucleotide sequence in coding region of five sequences was performed using Clustal X 1.83 (Supplemental Information 1). SNP events and transition (Ts) or transversion (Tv) in the plastomes were counted and positioned. In addition, SNPs were further classified into synonymous (S) and non-synonymous (N) substitutions using Mega 5.05 (Tamura et al., 2011). Then, a *Z* test (*P* < 0.05) was carried out by bootstrap method (1,000 replicates). The ancestral states were inferred using the ML method. The gene classification was according to Chang et al. (2006).

## Results & Discussion

### Assembly and features of cp genomes

**Figure 1:**
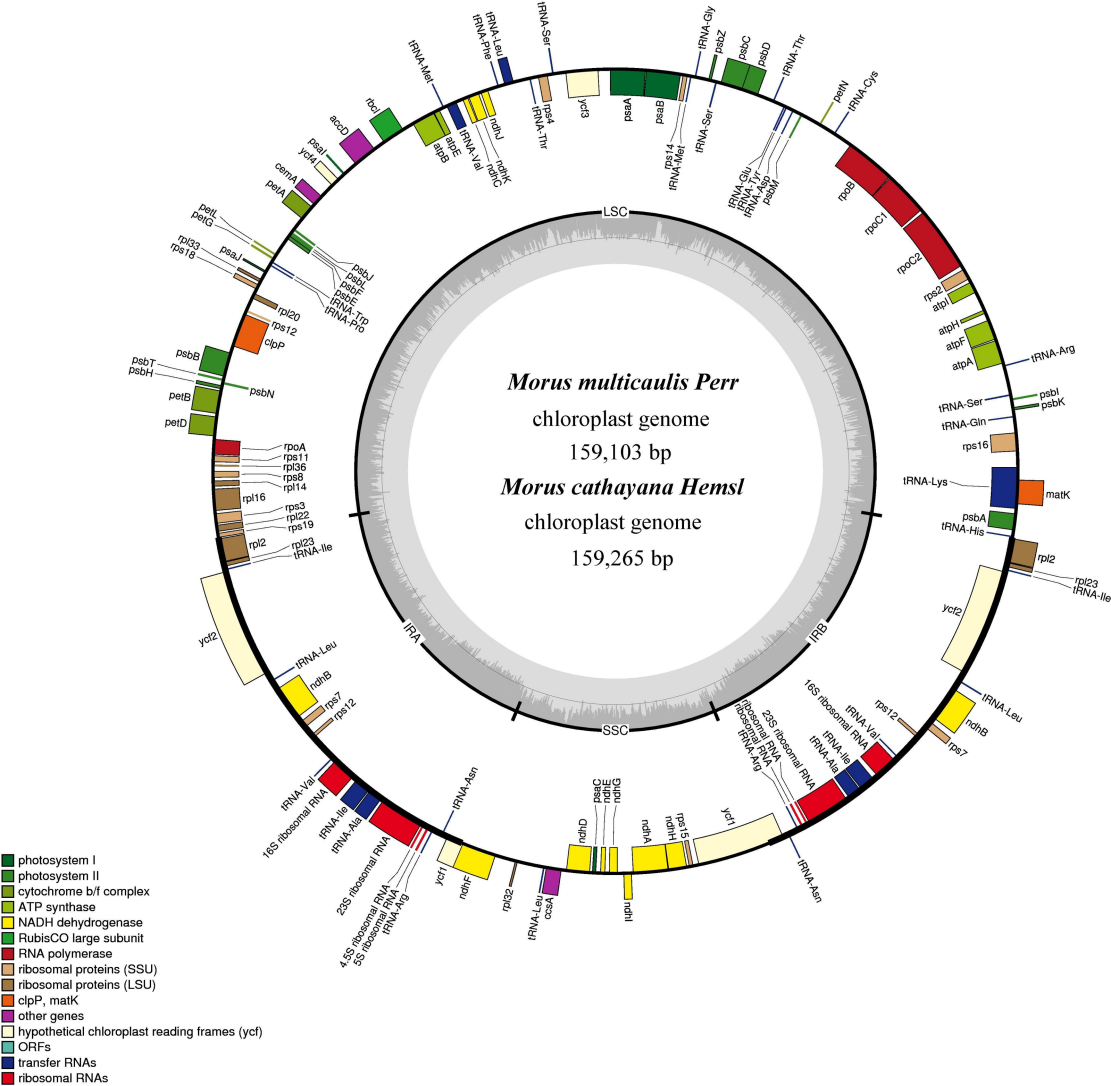
Gene map for *M. cathayana* and *M. multicaulis* plastomes. Genes lying outside the circle are transcribed in a clockwise direction, whereas genes inside are transcribed in a counterclockwise direction. Different colors denote known functional groups. The GC and AT contents of the genome are denoted by dashed darker and lighter gray in the inner circle. LSC, SSC, and IR indicate large single-copy, small single-copy, and inverted repeat regions, respectively.

The complete cp genomes of *M. cathayana* and *M. multicaulis* are both closed circular molecules of 159,265 and 159,103 bp (GenBank accession number: KU981118, KU981119), respectively (Fig. 1). Both cp genomes show the typical quadripartite structure of most angiosperms, and comprise a pair of IRs (25,639 bp for *M. cathayana* and 25,677 bp for *M. multicaulis*) separated by the LSC (88,143 bp for *M. cathayana* and 87,940 bp for *M. multicaulis*) and SSC (19,844 bp for *M. cathayana* and 19,809 bp for *M. multicaulis*) regions. The GC content of *M. cathayana* and *M. multicaulis* cp genomes is 36.16% and 36.19%, respectively. Similar to values reported for Rosaceae cp genomes (Su et al., 2014; Wang, Shi & Gao, 2013), the GC content of the five *Morus* spp. cp genomes is 36.16–36.37%, with uneven distribution within the cp genome: the GC content is highest in the IR (42.89–42.95%), intermediate in the LSC (33.77–34.12%), and lowest in the SSC region (29.20–29.35%) (Table 1).

The *M. cathayana* and *M. multicaulis* cp genomes both encode 132 predicted functional genes, of which 112 are unique genes, including 78 protein-coding genes (PCGs), 30 tRNA and four rRNA genes with 63,873 bp, 2,208 and 4,524 bp, respectively; 18 were duplicated in the IR region, including seven PCGs and seven tRNA and all rRNA genes (Fig. 1). Fifteen genes (10 PCGs and five tRNA genes) contain one intron, and three PCGs (*clpP*, *ycf3*, and *rps12*) have two introns. As found in most other green terrestrial plants, the maturase K (*matK*) gene in the *M. cathayana* and *M. multicaulis* cp genomes is located within the *trnK* intron; and *rps12* is a trans-spliced gene; the 5′-end exon is in the LSC region and the other two reside in the IR region separated by an intron. The four rRNA genes and two tRNA genes of *trnI* and *trnA* are clustered as 16S–*trnI*–*trnA*–23S–4.5S–5S in the IR region, the same as in the cp genome of *M. mongolica* and *M. indica* (Kong & Yang, 2016; Ravi et al., 2006) and most of other plants (Mardanov et al., 2008; Wang, Shi & Gao, 2013; Wu et al., 2014).

**Table 2:** SSRs in *M. cathayana* and *M. multicaulis* chloroplast genomes.

SSR	Size	NUMBER	LOCI
a	10	8	**3950**, **5052**,**590**,**29073**, **50058**, **68954**, **68987**, **114495(*ndhF*)**
	11	4	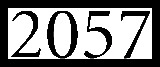 , 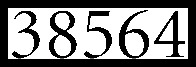 54282${}^{\underline{\mathrm{a12}}}$, **63153, 87796**, 116614${}^{\underline{\mathrm{a10}}}$
	12	2	13584${}^{\underline{\mathrm{a13}}}$, **84902**
	13	1	128314${}^{\underline{\mathrm{a14}}}$
	14	1	**74502**
	15	1	9565${}^{\underline{\mathrm{a11}}}$
	16	2	4799${}^{\underline{\mathrm{a13}}}$8984${}^{\underline{\mathrm{a15}}}$
t	10	17	**5231**, **9782**, 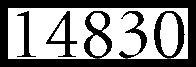 ,**24363**, **30678, 30944, 54323**, 55220, **57416(*atpB*), 62926, 67243, 69081, 71234, 74300**, 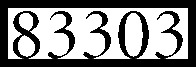 , **117117,122510, 30637(*ycf1*), 132394(*ycf1*)**
	11	8	**479**, 8575${}^{\underline{\mathrm{t10}}}$,**34221**, 57867${}^{\underline{\mathrm{t12}}}$,**59882**, 75017${}^{\underline{\mathrm{t13}}}$,**79022,131496(*ycf1*)**
	12	8	**12693**, 13275${}^{\underline{\mathrm{t13}}}$, 14182${}^{\underline{\mathrm{t10}}}$,**27623(*rpoB*)**, 68830${}^{\underline{\mathrm{t13}}}$, 69893${}^{\underline{\mathrm{t11}}}$,**72813,86135**
	13	3	9207${}^{\underline{\mathrm{t14}}}$, 52130${}^{\underline{\mathrm{t12}}}$,**128735**
	14	1	64181${}^{\underline{\mathrm{t13}}}$
	16	1	81690${}^{\underline{\mathrm{t13}}}$
	17	1	**49756**
	18(20)	1	87252${}^{\underline{\mathrm{t14}}}$, 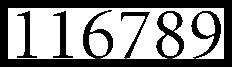
at	5	2	69153,**116007(*ndhF*)**
	6	1	**10796**
ta	6	3	**5495, 21235(*rpoC2*), 119074**
tc	6	1	**64908(*cemA*)**
aat	4	1	**128715**
ttc	4	1	**71261**
tat	4	1	50114
aaag	3	1	**135481**
aaat	3	3	**24057**, 38611, **47006**
atta	3	2	**33937, 116796**
attt	3	2	**14173, 62456**
tatt	3	1	**24394**
tctt	3	1	**111916**
ttat	3	1	118221
aagga	3	1	**14008(*atpF*)**
atata	3	1	117818
atttc	3	1	24297[tttct3]

**Notes.**

Parentheses, containing coding regions; boxed type, absent in *M. cathayana* but present in *M. multicaulis*; bolded type, absent in *M. multicaulis* but present in *M. cathayana*; underline and superscript, nucleotide length polymorphism; bracket, nucleotide content polymorphism, others are identical.

### Analyses of repetitive sequences

A total of 83 SSR loci, accounting for 973 bp, and 82 SSR loci, representing 949 bp in length, were detected in *M. cathayana* and *M. multicaulis* cp genomes (Table 2). Among these, there are 59 mono-, 7 di-, 3 tri-, 11 tetra-, and 3 penta-nucleotide repeats, respectively, in the *M. cathayana* cp genome; the corresponding numbers of these repeats in *M. multicaulis* are 63, 6, 2, 9, and 2. Mono-nucleotide repeats accounted for 71.1% and 76.8% of total SSRs in *M. cathayana* and *M. multicaulis*, respectively, similar to the levels found for *M. mongolica* and *M. indica* cp genomes, and the study on lower and higher plants (George et al., 2015). Six SSRs, including one di-nucleotide of (TC)_6_, one tri-nucleotide of (TTC)_4_, two tetra-nucleotides of (AAAG)_3_ and (TTCT)_3_, and two penta-nucleotides of (AAGGA)_3_ and (TTTCT)_3_ in *M. multicaulis* or (ATTTC)_3_ in *M. cathayana*, contain at least one C or G nucleotide, and the SSRs have a high AT content (97.5%). Most of the repeats are located in noncoding regions—i.e., intergenic spacers and introns—except for 10, which were found in seven coding genes of *ycf1*(3),* ndhF*(2), *rpoC2*, *rpoB*, *atpB*, *atpF* and *cemA*. The 10 SSRs in coding genes also occur in the *M. indica* and *M. mongolica* cp genomes. Comparison between *M. cathayana* and *M. multicaulis* revealed that 57 loci are identical, 19 exhibit length polymorphisms, six SSRs loci only exist in *M. cathayana*, and five SSRs loci only exist in *M. multicaulis*. One nucleotide content polymorphic of (TTTCT)_3_ and (ATTTC)_3_ exhibits in *M. multicaulis* and *M. cathayana*, respectively (Table 2).

A total of 14 long repeat sequences that are longer than 25 bp were identified in the five *Morus* spp. cp genomes, including one reverse, six forward and seven palindromic matches. Most of these repeats are located in intergenic spacers or introns, except for one 30 bp palindromic repeat, which is in the *trnS* genes (Table 3). Among all the long repeats, only four was detected in all the five cp genomes, which means that these repeats might create a diversity of cp genomes, and provide valuable information for phylogeny of genus *Morus* (Yang et al., 2014).

**Table 3:** Long repeat sequences in the chloroplast genome of five *Morus* species.

Type	Repeat size(bp)					Location	Region
	*M. cathayana*	*M. multicaulis*	*M. mongolica*	*M. indica*	*M. notabilis*		
P	51	–	–	–	–	IGS(*rpl2*-*trnH*; *rps19*-*rpl22*)	LSC
F	39	39	39	39	39	Intron(*ycf3*); IGS(*rps7*-*trnV*)	LSC; IRB
P	39	39	39	39	39	Intron(*ycf3*); IGS(*trnV*-*rps7*)	LSC; IRA
F	–	–	–	32	32	IGS(*trnT*-*trnL*)	LSC
F	–	31	31	31	31	IGS(*trnE*-*trnT*)	LSC
F	–	–	–	–	31	Intron(*clpP*)	LSC
P	30	30	30	30	30	*trnS*; *trnS*	LSC
P		30	–	30	–	Intron(*ndhA*)	SSC
R	28	–	26	26	27	IGS(*rps3*-*rpl22*)	LSC
P	28	28	28	–	28	IGS(*ccsA*-*ndhD*)	SSC
F	27	–	–	–	–	IGS(*rps7*-*trnV*)	IRA(IRB)
P	27	–	–	–	–	IGS(*rps7*-*trnV*; *trnV*-*rps7*)	IRA; IRB
P	26	26	26	26	26	IGS(*trnH*-*psbA*)	LSC
F	–	25	26	–	–	IGS(*ycf4*-*cemA*; *petD*-*rpoA*)	LSC

### Codon usage patterns in *M. cathayana* and *M. multicaulis* cp genomes, and comparison with other three *Morus* spp.

As an important indicator of CUB, the RSCU value is the frequency observed for a codon divided by the expected frequency (Sharp & Li, 1986). RSCU is close to 1.0 when all synonymous codons are used equally without any bias; RSCU is greater or less than 1 when synonymous codons are more or less frequent than expected (Gupta, Bhattacharyya & Ghosh, 2004). Codons ending with A and T have RSCU > 1 for *M. cathayana* and *M. multicaulis* cp genomes, indicating that they are used more frequently than synonymous codons, and may play major roles in the A+T bias of the entire cp genome. In terms of nucleotide composition, the most frequent codons are composed of T or A or their combination (i.e., ATT for Ile, AAA for Lys, AAT for Asn, and TTT for Phe); the least frequent codons have a high GC content (i.e., UGC for Cys, CGC and CGG for Arg, ACG for Thr, GCG for Ala, and CCG and CCC for Pro) (Table S1). Further analysis of the base composition at each codon position in the coding region revealed that the average GC content for GC1, GC2, and GC3 is lower than the AT content in the five *Morus* spp. cp genomes, and that the GC3 content is lower than the GC2 and GC1 content significantly (Table 4). The regular occurrence of A/T in the third codon position supports the previous finding that mutation towards A+T is a strong driving force for the cp genome.

**Table 4:** GC content of coding regions in the chloroplast genome of five *Morus* species.

Organism	CDS	Codons	GCc%	GC1%	GC2%	GC3%	GC3s%	ENc
*M. cathayana*	85	26599	37.29	45.13	37.42	29.3	26.28	49.28
*M. multicaulis*	85	26599	37.3	45.13	37.44	29.3	26.28	49.27
*M. indica*	84	26301	37.32	45.17	37.54	29.25	26.23	49.24
*M. mongolica*	84	26544	37.33	45.19	37.56	29.24	26.23	49.22
*M. notabilis*	84	26301	37.32	45.11	37.55	29.31	26.29	49.25

**Notes.**

CDS Coding sequence GCc GC content in coding regions GC1, GC2, GC3, GC3S GC content at first, second, third, and synonymous third codon positions, respectively ENc effective number of codons

**Figure 2:**
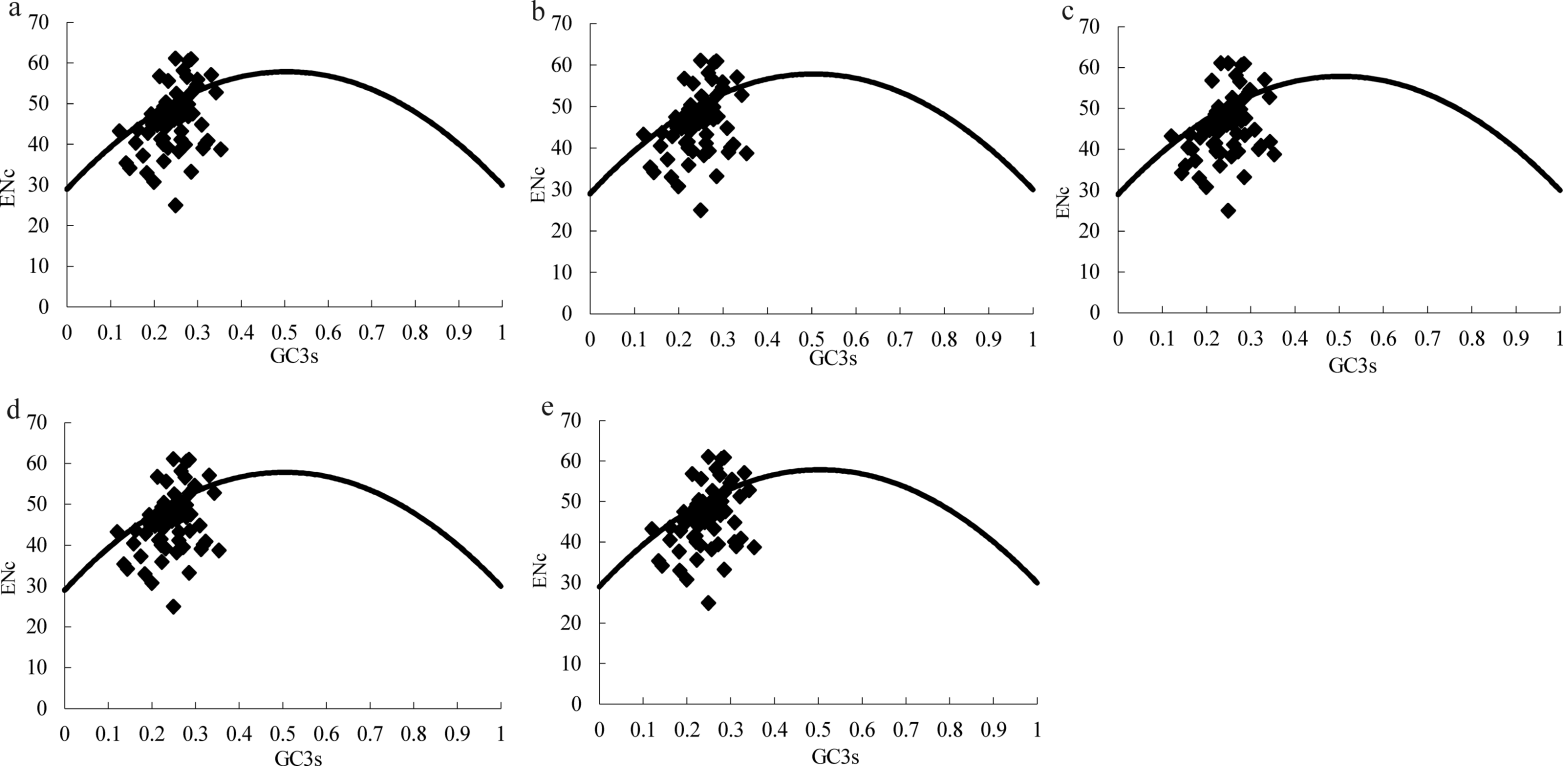
ENc plots for the chloroplast genome of five *Morus* species. Solid lines are expected ENc from GC3. (A) *M. cathayana*; (B) *M. multicaulis*; (C) *M. indica*; (D) *M. mongolica*; (E) *M. notabilis*.

The effective number of codons (ENc) is widely used as a measure of CUB, with range in 20–61, for genes with extreme bias using only one codon per amino acid or no bias using synonymous codons equally (Wright, 1990). GC3s is indicator of the level of nucleotide composition bias (Ahmad et al., 2013; Wright, 1990), and ENc plots (ENc vs GC3_S_) are a useful indicator of the factors affecting codon usage, as well as the force of mutation or other factors. Predicted values lie on or just below the expected curve when a gene’s codon usage is constrained only by the G+C mutation bias, and values are considerably below the curve when codon usage is subject to selection for codon optimization (Wright, 1990). Similar to the ENc plot for the Asteraceae family (Nie et al., 2013), the majority of genes among the five *Morus* spp. plastomes follow the standard curve, which indicates that the codon bias was mainly caused by nucleotide composition bias at the third position (GC3s) (Table 4, Fig. 2). In addition, there are several genes lying below the expected curve; this suggests that in addition to mutational bias, codon usage variation among genes can also be influenced by selection force (Sablok et al., 2011; Wright, 1990).

**Figure 3:**
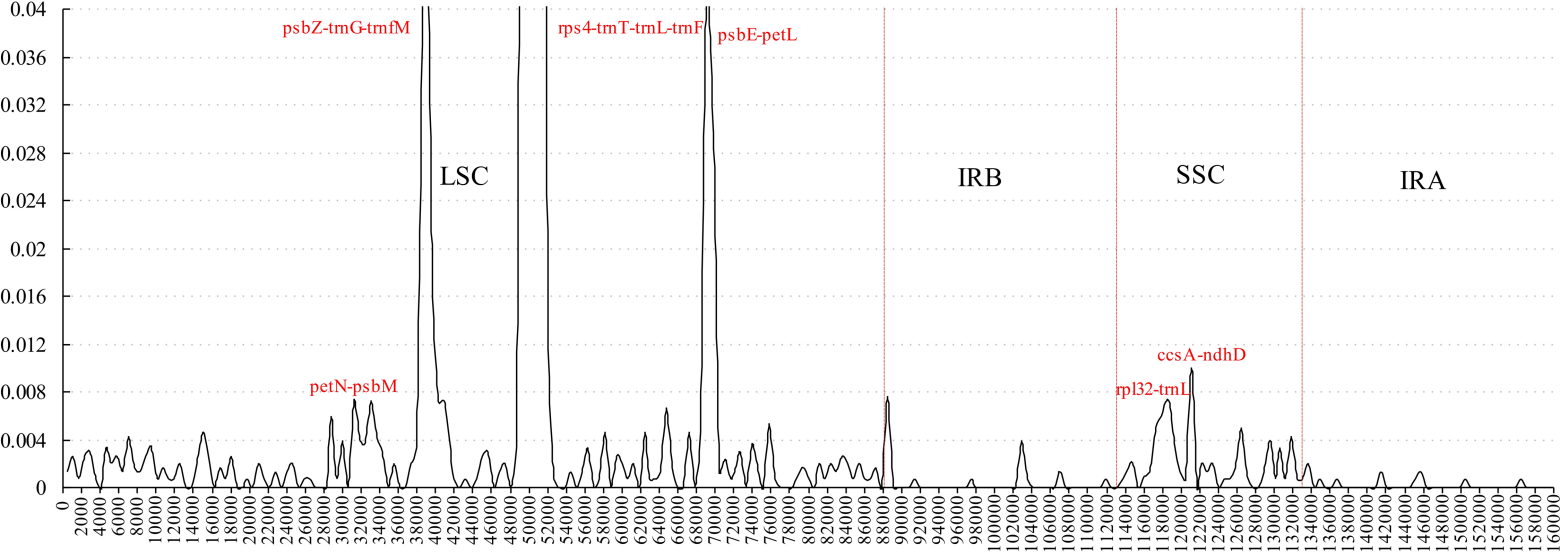
Sliding window analysis of the whole plastome for five *Morus* species (window length 600 bp, step size 200 bp). *X*-axis: position of the midpoint of a window; *Y*-axis: nucleotide diversity of each window.

**Table 5:** Transitions (Ts) and transversions (Tv) in the chloroplast genome of five *Morus* species.

	Ts		Tv			
	a < − >g	t < − >c	a < − >t	a < − >c	t < − >g	g < − >c
*M. notabilis*	13	22	4	9	18	4
*M. indica*	16	28	2	8	6	1
*M. mongolica*	–	–	–	1	–	1
*M. cathayana*	4	2	–	2	1	1
*M. multicaulis*	7	3	1	–	–	–
sum	40	55	7	20	25	7

**Table 6:** Synonymous (S) and non-synonymous (N) substitutions in chloroplast coding genes for five *Morus* species.

	Gene	*M. notabilis*		*M. mongolica*		*M. indica*		*M. cathayana*		*M. multicaulis*	
		S	N	S	N	S	N	S	N	S	N
Photosynthetic apparatus	*psaA*	1				1					
	*psaB*	1	1			21 ^−^	2		1		
	*psaI*									2	
	*psbA*								3	1	
	*psbC*	1									
	*psbD*					2					
	*psbE*					1					
	*psbK*		1								
	*psbZ*					1	2				
	*petD*					1					
	*petG*					1					
	*petL*						2				
	*ycf4*		1								
	**Total**	**3**	**3**	**0**	**0**	**28**	**6**	**0**	**4**	**3**	**0**
Photosynthetic metabolism	*atpA*	2									
	*atpB*					2	2			1	
	*atpE*	1								1	
	*ndhA*						1				1
	*ndhC*									1	
	*ndhD*	1	2				1				
	*ndhE*								1		
	*ndhF*	1	3	1			1	1			1
	*ndhI*	1									
	*ndhJ*		1								
	*rbcL*		6 ^+^							1	
	**Total**	**6**	**12**	**1**	**0**	**2**	**5**	**1**	**1**	**4**	**2**
Gene expression	*rpl14*	1	1								
	*rpl16*	1									
	*rpl20*	1				1					
	*rpl22*	1									
	*rpoA*	1							1		
	*rpoB*	1					1				
	*rpoC1*		2								
	*rpoC2*	2	3								
	*rps2*	1									
	*rps4*					2					
	*rps8*		2			2					
	*rps11*		1			1					
	*rps14*					3	5				
	*rps15*		1								
	*rps16*	1									
	*rps19*		1			2					
	**Total**	**10**	**11**	**0**	**0**	**11**	**6**	**0**	**1**	**0**	**0**
Other genes	*accD*		1	1					1		
	*cemA*		1								
	*ccsA*	1									
	*matK*	2	3			1				1	
	*ycf1*	5	11 ^+^				2		2		1
	*ycf2*		1								
	**Total**	8	17	1	0	1	2	0	3	1	1
	**Sum**	**27**	**43**	**2**	**0**	**42**	**19**	**1**	**9**	**8**	**3**

**Notes.**

+ positive selection − negative selection

### Sequence divergence among five *Morus* spp. cp genomes

Comparative analysis of sequence divergence among the five *Morus* spp. cp genomes revealed nucleotide variability (*Pi*) values in the range 0–0.28533 with average of 0.00432, indicating that the differences among the five cp genomes is small. However, nine intergenic regions (*trnT*–*trnL*, *petN–psbM*, *psbZ*–*trnG*, *trnG*–*trnfM*, *rps4*–*trnT*, *psbE*–*petL*, *rpl32*–*trnL*, *trnL*–*trnF* and *ccsA*–*ndhD*) and one intron (trnL) are highly variable, with much higher values (*Pi* > 0.008) than other regions (Fig. 3). All the 10 loci are in single-copy regions, not IR regions. These regions with highly variable loci are not random but are clustered in ‘hot spots’ (Shaw et al., 2007; Worberg et al., 2007). It has been reported that *rpl32–trnL* is particularly highly variable in *Machilus* and *Solanum* plastomes, and in spermatophyte (Dong et al., 2012; Dong et al., 2015; Sarkinen & George, 2013; Song et al., 2015), and that *trnL*–*trnF*, *trnT*–*trnL*, *rps4*–*trnT*, and the *trnL* intron are highly variable between two species of genus *Morus* L. (Kong & Yang, 2016). The *petN–psbM* intergenic region was reported as part of the *trnC–trnD* intergenic region, and has been used in lower-level phylogenetic studies in flowering plants (Lee & Wen, 2004). The *petN–psbM* region and the *clpP* intron were successfully used in a study of phylogenetic relationships in peach species (Dong et al., 2012). In the present study, four rarely reported highly variable loci, *psbZ*–*trnG*, *trnG*–*trnfM*, *psbE*–*petL*, and *ccsA*–*ndhD*, were found in *Morus* plastomes. Phylogenetic studies at the species level in *Morus* have not been carried out using highly variable regions, and our analysis provides a basis for further phylogenetic study of *Morus* using these highly variable regions.

### Numbers and pattern of SNP mutations

As the most abundant type of mutation, we investigated SNPs in gene coding regions of five *Morus* spp. cp genomes. There were 154 SNPs detected, including 95 Ts, 59 Tv, and 74 N and 80 S substitutions (Tables 5 and 6). Among the Tv, 45 are GC content changes, 14 are between A and T, and between G and C (Table 5). The Kn, Ks and their ratio are indicators of the rates of evolution and natural selection (Yang & Nielsen, 2000). Relative to the reference ancestral states, 74 non-synonymous substitutions was observed in 28 of 79 protein-coding regions in five *Morus* spp. (Table 6). The photosynthetic apparatus gene *psaB* of *M. indica* share extra synonymous substitution sites, while *ycf1*, photosynthetic metabolism gene *rbcL* of *M. notabilis* and gene expression gene *rps14* of *M. indica* has more non-synonymous than synonymous substitution sites, suggesting a relatively high evolution rate for these genes, as for *M. yunnanensis* and *M. balansae* (Song et al., 2015). A *Z* test can be used to detect positive selection by comparing the relative abundance of synonymous and non-synonymous substitutions within the gene sequences, the results of the *Z* test for each gene showed that *rbcL* and *ycf1* genes of *M. notabilis* exist positive selection, and *psaB* of *M. indica* exists negative selection, which means that the genes of *Morus* chloroplast might exert different evolutionary pressures. For all of these substitutions, 74.03% and 25.32% of the SNP sites are in LSC and SSC regions, respectively, while only one SNP site is in *M. indica* IR region, consistent with the more conservative characteristics of IR compared to LSC and SSC regions (Dong et al., 2014; Jo et al., 2011).

##  Supplemental Information

10.7717/peerj.3037/supp-1 Table S1 Codon usage and codon–anticodon recognition pattern for tRNA in *M. cathayana* and *M. multicaulis* chloroplast genomes

10.7717/peerj.3037/supp-2 Supplemental Information 1 Thenucleotide matrices used in the analysis

## Additional Information and Declarations

### DNA Deposition

The following information was supplied regarding the deposition of DNA sequences: GenBank accession number: KU981118, KU981119.

### Data Availability

The following information was supplied regarding data availability: BioProject ID: PRJNA326788; PRJNA326736
