# Supplementary material for: The complete chloroplast genome sequence of Morus cathayana and Morus multicaulis, and comparative analysis within genus Morus L
Source: PeerJ. 2017 Mar 8;5:e3037. doi: 10.7717/peerj.3037 (PMC5345388; doi:10.7717/peerj.3037)
Supplement: Table S1 [file peerj-05-3037-s001.docx]

Table S1 Codon usage and codon–anticodon recognition pattern for tRNA in *M. cathayana* and *M. multicaulis* chloroplast genomes

| AA | codon | *M. cathayana* | | *M. multicaulis* | | tRNA | AA | codon | *M. cathayana* | | *M. multicaulis* | | tRNA |
| --- | --- | --- | --- | --- | --- | --- | --- | --- | --- | --- | --- | --- | --- |
|  |  | Count | RSCU | Count | rscu |  |  |  | Count | RSCU | Count | rscu |  |
| Phe | UUU(F) | 1012 | 1.33 | 1012 | 1.33 |  | Tyr | UAU(Y) | 812 | 1.62 | 812 | 1.62 |  |
|  | UUC(F) | 512 | 0.67 | 512 | 0.67 | trnF-GAA |  | UAC(Y) | 191 | 0.38 | 191 | 0.38 | trnY-GUA |
| Leu | UUA(L) | 896 | 1.88 | 897 | 1.89 | trnL-UAA | Ter | UAA(*) | 44 | 1.55 | 44 | 1.55 |  |
|  | UUG(L) | 572 | 1.2 | 572 | 1.2 | trnL-CAA |  | UAG(*) | 24 | 0.85 | 23 | 0.81 |  |
|  | CUU(L) | 616 | 1.3 | 616 | 1.3 |  | His | CAU(H) | 490 | 1.54 | 490 | 1.54 |  |
|  | CUC(L) | 192 | 0.4 | 192 | 0.4 |  |  | CAC(H) | 147 | 0.46 | 147 | 0.46 | trnH-GUG |
|  | CUA(L) | 391 | 0.82 | 391 | 0.82 | trnL-UAG | Gln | CAA(Q) | 724 | 1.55 | 724 | 1.55 | trnQ-UUG |
|  | CUG(L) | 186 | 0.39 | 184 | 0.39 |  |  | CAG(Q) | 208 | 0.45 | 208 | 0.45 |  |
| Ile | AUU(I) | 1116 | 1.45 | 1115 | 1.45 |  | Asn | AAU(N) | 1018 | 1.53 | 1017 | 1.54 |  |
|  | AUC(I) | 447 | 0.58 | 447 | 0.58 | trnI-GAU |  | AAC(N) | 310 | 0.47 | 308 | 0.46 | trnN-GUU |
|  | AUA(I) | 747 | 0.97 | 747 | 0.97 |  | Lys | AAA(K) | 1070 | 1.49 | 1070 | 1.49 | trnK-UUU |
| Met | AUG(M) | 629 | 1 | 630 | 1 | trnM-CAU | | AAG(K) | 368 | 0.51 | 368 | 0.51 |  |
| Val | GUU(V) | 512 | 1.44 | 512 | 1.44 |  | Asp | GAU(D) | 866 | 1.62 | 865 | 1.61 |  |
|  | GUC(V) | 167 | 0.47 | 167 | 0.47 | trnV-GAC |  | GAC(D) | 206 | 0.38 | 207 | 0.39 | trnD-GUC |
|  | GUA(V) | 556 | 1.56 | 555 | 1.56 | trnV-UAC | Glu | GAA(E) | 1066 | 1.52 | 1066 | 1.52 | trnE-UUC |
|  | GUG(V) | 187 | 0.53 | 187 | 0.53 |  |  | GAG(E) | 340 | 0.48 | 341 | 0.48 |  |
| Ser | UCU(S) | 571 | 1.7 | 573 | 1.7 |  | Cys | UGU(C) | 237 | 1.53 | 237 | 1.53 |  |
|  | UCC(S) | 329 | 0.98 | 328 | 0.98 | trnS-GGA |  | UGC(C) | 72 | 0.47 | 72 | 0.47 | trnC-GCA |
|  | UCA(S) | 419 | 1.25 | 418 | 1.24 | trnS-UGA | Ter | UGA(*) | 17 | 0.6 | 18 | 0.64 |  |
|  | UCG(S) | 175 | 0.52 | 175 | 0.52 |  | Trp | UGG(W) | 459 | 1 | 459 | 1 | trnW-CCA |
| Pro | CCU(P) | 418 | 1.54 | 418 | 1.53 |  | Arg | CGU(R) | 342 | 1.3 | 342 | 1.3 | trnR-ACG |
|  | CCC(P) | 196 | 0.72 | 197 | 0.72 |  |  | CGC(R) | 104 | 0.39 | 104 | 0.39 |  |
|  | CCA(P) | 319 | 1.17 | 319 | 1.17 | trnP-UGG |  | CGA(R) | 354 | 1.34 | 354 | 1.34 |  |
|  | CCG(P) | 155 | 0.57 | 156 | 0.57 |  |  | CGG(R) | 104 | 0.39 | 104 | 0.39 |  |
| Thr | ACU(T) | 552 | 1.66 | 553 | 1.66 |  | Ser | AGU(S) | 395 | 1.18 | 394 | 1.17 |  |
|  | ACC(T) | 241 | 0.72 | 241 | 0.72 | trnT-GGU |  | AGC(S) | 128 | 0.38 | 129 | 0.38 | trnS-GCU |
|  | ACA(T) | 390 | 1.17 | 390 | 1.17 | trnT-UGU | Arg | AGA(R) | 496 | 1.88 | 496 | 1.88 | trnR-UCU |
|  | ACG(T) | 147 | 0.44 | 147 | 0.44 |  |  | AGG(R) | 180 | 0.68 | 180 | 0.68 |  |
| Ala | GCU(A) | 624 | 1.82 | 625 | 1.83 |  | Gly | GGU(G) | 588 | 1.33 | 589 | 1.33 |  |
|  | GCC(A) | 206 | 0.6 | 206 | 0.6 |  |  | GGC(G) | 180 | 0.41 | 180 | 0.41 |  |
|  | GCA(A) | 392 | 1.15 | 391 | 1.14 | trnA-UGC |  | GGA(G) | 728 | 1.65 | 728 | 1.64 | trnG-UCC |
|  | GCG(A) | 147 | 0.43 | 147 | 0.43 |  |  | GGG(G) | 274 | 0.62 | 274 | 0.62 |  |

Notes.

*: stop codon
